# Prevalence of and characteristics associated with operative vaginal birth at Mizan-Tepi University Teaching Hospital

**DOI:** 10.1093/inthealth/ihaa024

**Published:** 2020-06-01

**Authors:** Margo S Harrison, Biruk Teshome, Tewodros Liyew, Ephrem Kirub, Andrea Jimenez-Zambrano, Margaret Muldrow, Teklemariam Yarinbab

**Affiliations:** University of Colorado School of Medicine, Aurora, Colorado, USA; Mizan-Tepi University Teaching Hospital, Aman, Bench Maji Zone, Ethiopia; Mizan-Tepi University Teaching Hospital, Aman, Bench Maji Zone, Ethiopia; Mizan-Tepi University Teaching Hospital, Aman, Bench Maji Zone, Ethiopia; University of Colorado School of Medicine, Aurora, Colorado, USA; Village Health Partnership, Denver, Colorado, USA; Mizan-Tepi University Teaching Hospital, Aman, Bench Maji Zone, Ethiopia

**Keywords:** Ethiopia, operative vaginal birth

## Abstract

**Background:**

To observe prevalence, characteristics and outcomes associated with operative vaginal birth (OVB).

**Methods:**

We compared spontaneous vaginal birth with OVB.

**Results:**

Of 993 women, 759 (76.4%) experienced vaginal birth; 716 were spontaneous (94.3%), 14 (1.8%) underwent forceps-assisted birth and 29 (3.8%) had vacuum assistance. In a multivariable model of OVB (forceps and vacuum), compared with a midwife, general practitioners (OR 5.6, *p* = 0.04) and integrated emergency surgical officers (OR 42.8, *p* = 0.001) were more likely to attend. Women experiencing OVB were more likely to receive local anesthesia (OR 3.0, *p* = 0.009).

**Conclusion:**

OVB is used sparingly but safely at Mizan-Tepi University Teaching Hospital.

## Introduction

Operative vaginal birth (OVB), which refers to forceps- and vacuum-assisted vaginal birth, in appropriate candidates, can be an alternative to cesarean birth.^1^ It is often used in settings where the uterine ‘power’ or strength of contractions to move a fetus through the birth canal is not sufficient.^2^ Recent literature has shown that forceps- and vacuum-assisted vaginal births are uncommon (less than 5%) in low- and middle-income countries and rates of use may be declining.^3^ We wanted to observe the use of these obstetric methods at Mizan-Tepi University Teaching Hospital (MTUTH) in Ethiopia to determine characteristics and outcomes associated with and prevalence of OVB in this setting.

## Methodology

We executed a hospital-based, prospective, cross-sectional study at MTUTH, in the Southern Nations, Nationalities and People's Region of Ethiopia to observe service delivery in order to design and guide future quality improvement interventions and initiatives. We followed a convenience sample of 1000 women (those who conveniently presented to the facility to give birth after 28 completed weeks of gestation) at MTUTH between 6 May and 21 October 2019. Through a combination of chart review and structured interview at admission, delivery and discharge, three physicians collected de-identified data on paper forms, which was then entered into REDCap (an electronic data capture system) after quality checking of the paper forms was complete. The rationale for performing this work at MTUTH is that it is the only major referral facility in the Bench Maji Zone and, given a catchment area of 2.5 million people, we wanted to ensure that high-quality care is being delivered.

Bivariate comparisons of sociodemographic, obstetric, birth and pregnancy outcomes of women experiencing vaginal vs OVB were performed using STATA software version 15.2 (StataCorp LP, College Station, TX, USA). Fisher's exact, χ^2^ and Kruskal-Wallis tests were performed depending on the variables. All covariates significant to *p* < 0.05 were included in a multivariable logistic regression to determine which covariates were independently associated with cesarean birth. Subsequently, individual logistic regressions, adjusted for covariates significant in the first multivariable model to *p* < 0.05, were executed to observe the association of OVB with a number of maternal and neonatal outcomes.

## Results

Table 1A shows the characteristics of the overall study population as well as those of women delivering by spontaneous vaginal birth compared with OVB. Overall, the population was young (median age 24 y, IQR range 20–28 y), had experienced some amount of schooling (77.2%) and had a religious affiliation (all patients); the majority were married (96.2%), lived in an urban setting (51.2%) and had a median of four prenatal visits (IQR 3–5).

**Table 1.** Bivariate comparisons and multivariable modeling of women experiencing spontaneous vaginal birth with those who gave birth with vacuum assistance

(A) Bivariate comparisons of women who experienced unassisted vaginal birth compared with those who underwent assisted vaginal birth (operative vaginal delivery)				
Characteristic	Total N = 759	Unassisted vaginal birth (n = 716, 94.3%)	Assisted vaginal birth (n = 43, 5.7%)	*p*-value**
Sociodemographic				
Age, y (median [IQR])	24 [20–28]	22 [20–29]	24 [20–28]	0.62^a^
Missing	1 (0.1%)	1 (0.1%)	0 (0.0%)	
Education				0.53^b^
Unable to read and write	173 (22.8%)	160 (22.3%)	13 (30.2%)	
Read and write only	46 (6.1%)	44 (6.2%)	2 (4.7%)	
Primary school	302 (39.8%)	288 (40.2%)	14 (32.6%)	
Secondary school	106 (14.0%)	102 (14.3%)	4 (9.3%)	
Higher education	131 (17.3%)	121 (16.9%)	10 (23.3%)	
Missing	1 (0.1%)	1 (0.1%)	0 (0.0%)	
Religion				0.78^b^
Muslim	79 (10.4%)	75 (10.5%)	4 (9.3%)	
Orthodox Christian	256 (33.7%)	239 (33.4%)	17 (39.5%)	
Catholic Christian	0 (0.0%)	0 (0.0%)	0 (0.0%)	
Protestant	421 (55.5%)	399 (55.7%)	22 (51.2%)	
Jehovah's Witness	2 (0.3%)	2 (0.3%)	0 (0.0%)	
Missing	1 (0.1%)	1 (0.1%)	0 (0.0%)	
Relationship status				0.58^b^
Single	22 (2.9%)	20 (2.8%)	2 (4.7%)	
Not single	730 (96.2%)	689 (96.2%)	41 (95.3%)	
Missing	7 (0.9%)	7 (1.0%)	0 (0.0%)	
Woreda				0.03^c^
Urban	389 (51.2%)	360 (50.3%)	29 (67.4%)	
Rural	370 (48.8%)	356 (49.7%)	14 (32.6%)	
Missing	0 (0.0%)	0 (0.0%)	0 (0.0%)	
Number of prenatal visits				0.52^a^
Median (IQR)	4 [3–5]	4 [3–5]	4 [3–6]	
Missing	5 (0.7%)	5 (0.7%)	0 (0.0%)	
Antepartum, labor and delivery				
Parity				0.12^b^
0	325 (42.8%)	302 (42.2%)	23 (53.5%)	
1	199 (26.2%)	189 (26.4%)	10 (23.3%)	
2	120 (15.8%)	118 (16.5%)	2 (4.7%)	
⩾3	114 (15.0%)	106 (14.8%)	8 (18.6%)	
Missing	1 (0.1%)	1 (0.1%)	0 (0.0%)	
Number of prior cesarean births				0.34^b^
0	741 (97.6%)	700 (97.8%)	41 (95.4%)	
1	15 (2.0%)	13 (1.8%)	2 (4.7%)	
2	1 (0.1%)	1 (0.1%)	0 (0.0%)	
Missing	2 (0.3%)	2 (0.3%)	0 (0.0%)	
Interpregnancy interval, mo (IQR)	60 [36, 84]	60 [36, 84]	42 [36, 60]	0.20^b^
Missing	0 (0.0%)	0 (0.0%)	0 (0.0%)	
Labor onset				0.10^b^
Not applicable	3 (0.4%)	2 (0.3%)	1 (2.3%)	
Spontaneous	663 (87.4%)	628 (87.7%)	35 (81.4%)	
Induced/augmented	92 (12.1%)	85 (11.9%)	7 (16.3%)	
Missing	1 (0.1%)	1 (0.1%)	0 (0.0%)	
Transferred				0.007^c^
No	416 (54.8%)	401 (56.0%)	15 (2.3%)	
Yes	342 (45.1%)	628 (43.9%)	28 (65.1%)	
Missing	1 (0.1%)	1 (0.1%)	0 (0.0%)	
Cervical dilation on admission (cm)				0.02^a^
Median (IQR)	4 [2–7]	3 [2–7]	4.5 [3–10]	
Missing	14 (1.8%)	13 (1.8%)	1 (2.3%)	
Duration of labor, h				0.28^b^
Not applicable	6 (0.8%)	6 (0.8%)	0 (0.0%)	
<12s	424 (55.9%)	404 (56.4%)	20 (46.5%)	
12–24	304 (40.0%)	284 (39.7%)	20 (46.5%)	
⩾24	25 (3.3%)	22 (3.1%)	3 (7.0%)	
Missing	0 (0.0%)	0 (0.0%)	0 (0.0%)	
Antepartum hemorrhage				0.51^b^
No	746 (98.3%)	704 (98.3%)	42 (97.7%)	
Yes	12 (1.6%)	11 (1.5%)	1 (2.3%)	
Missing	1 (0.1%)	1 (0.1%)	0 (0.0%)	
Chorioamnionitis				1.0^b^
No	755 (99.5%)	712 (99.4%)	43 (100.0%)	
Yes	4 (0.5%)	4 (0.6%)	0 (0.0%)	
Missing	0 (0.0%)	0 (0.0%)	0 (0.0%)	
Antepartum pre-eclampsia/eclampsia/chronic hypertension				1.0^b^
No	724 (95.4%)	683 (95.4%)	41 (95.4%)	
Yes	34 (4.5%)	32 (4.5%)	2 (4.6%)	
Missing	1 (0.1%)	1 (0.1%)	0 (0.0%)	
Anesthesia				<0.001^b^
No	671 (88.4%)	643 (89.8%)	28 (65.1%)	
Yes, local	87 (11.5%)	73 (10.2%)	14 (32.6%)	
Yes, spinal	1 (0.1%)	0 (0.0%)	1 (2.3%)	
Missing	0 (0.0%)	0 (0.0%)	0 (0.0%)	
Delivery provider				<0.001^b^
Midwife	729 (96.0%)	702 (98.0%)	27 (62.8%)	
General practitioner	9 (1.2%)	7 (1.0%)	2 (4.7%)	
Integrated emergency surgical officer	20 (2.6%)	6 (0.8%)	14 (32.6%)	
Obstetrician	1 (0.1%)	1 (0.1%)	0 (0.0%)	
Missing	0 (0.0%)	0 (0.0%)	0 (0.0%)	
Gestational age, wk (median [IQR])	38 [38–40]	38 [38–40]	38 [38–40]	0.66^a^
Missing	2 (2.5%)	2 (2.5%)	0 (0.0%)	
Birth weight, g				0.76^b^
<2500	57 (7.5%)	55 (7.7%)	2 (4.6%)	
$\quad \ge $2500	671 (88.4%)	15 (88.3%)	33 (90.8%)	
Missing	31 (4.1%)	29 (4.1%)	2 (4.6%)	
Baby gender				0.12^c^
Male	401 (52.8%)	373 (54.4%)	28 (66.7%)	
Female	327 (43.1%)	313 (45.6%)	14 (33.3%)	
Missing	31 (4.1%)	0 (0.0%)	0 (0.0%)	
Postpartum complications				
MATERNAL				
Postpartum hemorrhage				1.0^b^
No	755 (99.5%)	712 (99.4%)	43 (100.0%)	
Yes	4 (0.5%)	4 (0.6%)	0 (0.0%)	
Missing	0 (0.0%)	0 (0.0%)	0 (0.0%)	
Uterotonics				1.0^b^
No	756 (99.6%)	713 (99.6%)	43 (100.0%)	
Yes	2 (0.3%)	2 (0.3%)	0 (0.0%)	
Missing	1 (0.1%)	1 (0.1%)	0 (0.0%)	
Blood transfusion				1.0^b^
No	755 (99.5%)	712 (99.4%)	43 (100.0%)	
Yes	4 (0.5%)	4 (0.6%)	0 (0.0%)	
Missing	0 (0.0%)	0 (0.0%)	0 (0.0%)	
Postpartum antibiotics				*0.07* *^b^*
No	740 (97.5%)	700 (97.8%)	40 (93.0%)	
Yes	17 (2.2%)	14 (2.0%)	3 (7.0%)	
Missing	2 (0.3%)	2 (0.2%)	0 (0.0%)	
Perineal tear				-
No	758 (99.9%)	715 (99.9%)	43 (100.0%)	
Yes	0 (0.0%)	0 (0.0%)	0 (0.0%)	
Missing	1 (0.1%)	1 (0.1%)	0 (0.0%)	
Days hospitalized (IQR)	1 [1–1]	1 [1–1]	1 [1–1]	0.24^a^
Missing	3 (0.4%)	0 (0.0%)	0 (0.0%)	
NEONATAL OUTCOMES				
5-min Apgar score median (IQR)	9 [8–9]	9 [8–9]	8 [7–9]	<0.001^a^
Missing	30 (4.0%)	29 (4.1%)	1 (2.3%)	
Fetal status at delivery				*0.09* *^b^*
Alive	702 (99.9%)	663 (92.7%)	39 (90.7%)	
Fresh stillbirth	15 (0.0%)	12 (1.7%)	3 (7.0%)	
Macerated stillbirth	11 (0.1%)	11 (1.5%)	0 (0.0%)	
Missing	31 (4.1%)*	29 (4.1%)	1 (2.3%)	
Bag and mask resuscitation				*0.09* *^b^*
No	746 (98.3%)	705 (98.4%)	41 (95.4%)	
Yes	9 (1.2%)	7 (1.0%)	2 (4.6%)	
Missing	4 (0.5%)	4 (0.6%)	0 (0.0%)	
Oxygen				0.23^b^
No	740 (97.5%)	699 (97.6%)	41 (95.4%)	
Yes	16 (2.1%)	14 (2.0%)	2 (4.6%)	
Missing	3 (0.4%)	3 (0.4%)	0 (0.0%)	
Continuous positive airway pressure (CPAP)				1.0^b^
No	750 (98.8%)	708 (98.9%)	42 (97.7%)	
Yes	5 (0.7%)	5 (0.7%)	0 (0.0%)	
Missing	4 (0.5%)	3 (0.4%)	1 (2.3%)	
Neonatal antibiotics				0.18^b^
No	727 (95.8%)	687 (96.0%)	40 (93.0%)	
Yes	26 (3.4%)	23 (3.2%)	3 (7.0%)	
Missing	6 (0.8%)	6 (0.8%)	0 (0.0%)	
Blood transfusion				0.15^b^
No	750 (99.0%)	709 (95.4%)	41 (98.8%)	
Yes	3 (0.3%)	2 (2.3%)	1 (0.4%)	
Missing	6 (0.7%)	5 (2.3%)	1 (2.8%)	
Neonatal demise				0.04^b^
Dead	35 (4.6%)	30 (4.2%)	5 (11.6%)	
Alive	720 (94.9%)	682 (95.3%)	38 (88.4%)	
Missing	4 (0.5%)	0 (0.6%)	0 (0.0%)	
Days hospitalized [IQR]	1 [1–1]	1 [1–1]	1 [1–1]	0.52^a^
Missing	13 (1.7%)	11 (1.5%)	2 (4.7%)	
(B) Multivariable model of characteristics associated with assisted vaginal birth				
Characteristic	AOR	CI	*p*-value	
Living in an urban area	1.0	0.4 to 2.3	0.95	
Transferred from another facility	1.7	0.7 to 3.9	0.21	
Cervical dilation on admission, cm	1.0	0.9 to 1.2	0.57	
Compared with a midwife attendant				
General practitioner	5.6	1.0 to 30.0	0.04	
Integrated emergency surgical officer	42.8	13.8 to 132.3	<0.001	
Compared with no anesthesia				
Local anesthesia	3.0	1.3 to 7.0	0.009	
(C) Multivariable model of individual adjusted logistic regressions of association of operative vaginal birth with maternal and neonatal outcomes significant in bivariate comparisons (Table 1)^d^				
Characteristic	AOR	CI	*p*-value	
Maternal postpartum antibiotic administration	2.0	0.4 to 9.8	0.38	
Apgar score ≥ 7	0.6	0.2 to 1.8	0.40	
Live birth occurred	0.9	0.2 to 4.1	0.86	
Infant needed resuscitation with bag and mask	1.7	0.2 to 3.7	0.64	
Infant was alive at discharge from hospital	0.6	0.2 to 2.1	0.43	

a Kruskal-Wallis test.

b Fisher's Exact test.

c χ^2^ test.

d Adjusted for delivery attendant and type of anesthesia.

* Missing data on one fetal status re: mode of delivery.

** Please note that all tests of covariates in bivariate comparisons do not include missing data.

Women who delivered by OVB were more likely to be from an urban area (67.4% vs 50.3%, *p* = 0.03, χ^2^ test), to have been transferred to MTUTH from another facility (65.1% vs 43.9%, *p* = 0.007, χ^2^ test), to have had greater cervical dilation on admission (4.5 vs 3 cm, *p* = 0.02, Kruskal-Wallis test), to receive local or spinal anesthesia (34.9% vs 10.2%, *p* < 0.001, Fisher's exact test) and to be delivered by a physician or integrated emergency and surgical officer than a midwife (37.3% vs 1.9%, *p* < 0.001, Fisher's exact test). Adverse outcomes that were more likely to result after a woman delivered by OVB included: a lower 5-min Apgar score (8 vs 9, *p* < 0.001, χ^2^ test) and a higher prevalence of neonatal demise (11.6% vs 4.2%, *p* = 0.04, Fisher's exact test). All tests were performed on available data and missing data were excluded from the comparison.

Table 1B displays a multivariable logistic regression of antepartum characteristics associated with OVB. In a multivariate logistic regression predicting OVB, general practitioners (adjusted OR [aOR] 5.6) and surgical officers (aOR 42.8) were more likely to perform OVD procedures compared with midwives (*p* = 0.04 and *p* = 0.001, respectively; Table 1B). Odds of use of local anesthesia was also higher in women giving birth with assistance (aOR 3.0, *p* = 0.009).

To analyze if there was any association of OVB and adverse pregnancy outcomes, we then performed individual logistic regressions of OVB to test the association with various maternal and neonatal outcomes, adjusted for delivery attendant and anesthesia use. As shown in Table 1C, none of these individual models were significant.

## Discussion

The overall prevalence of OVB at MTUTH is 5.7%, which is consistent with usage globally, regardless of development index.^3^ The obstetric skill is provided by higher level providers (general practitioners and integrated emergency and surgical officers) and is often associated with the use of local anesthesia, presumably for perineal tear repair, but is not associated with advanced tearing in this setting.^2^ No other adverse maternal and neonatal outcomes were found to be more prevalent after OVB compared with routine vaginal birth, suggesting that the procedure is being performed without undue harm. The implication of this analysis is that with global cesarean birth rates rising, OVB may be an alternative mode of delivery that can reduce unnecessary cesarean birth.^4^ The experience at MTUTH, a low-resource setting, suggests that the procedure can be used by mid-level non-physician providers without undue harm to women and infants, which is a reassuring finding related to OVB.
